# Regulation of GVBD in mouse oocytes by miR-125a-3p and Fyn kinase through modulation of actin filaments

**DOI:** 10.1038/s41598-017-02071-x

**Published:** 2017-05-22

**Authors:** Hadas Grossman, Efrat Har-Paz, Natalie Gindi, Mattan Levi, Irit Miller, Nava Nevo, Dalia Galiani, Nava Dekel, Ruth Shalgi

**Affiliations:** 1 Department of Cell and Developmental Biology, Sackler Faculty of Medicine, Tel-Aviv University, Ramat-Aviv, Tel-Aviv 69978 Israel; 2 Department of Biological Regulation, Weizmann Institute of Science, Rehovot, 76100 Israel

## Abstract

Meiotically arrested oocytes are characterized by the presence of the nuclear structure known as germinal-vesicle (GV), the breakdown of which (GVBD) is associated with resumption of meiosis. Fyn is a pivotal factor in resumption of the first meiotic division; its inhibition markedly decreases the fraction of oocytes undergoing GVBD. Here, we reveal that in mouse oocytes Fyn is post-transcriptionally regulated by miR-125a-3p. We demonstrate that in oocytes resuming meiosis miR-125a-3p and Fyn exhibit a reciprocal expression pattern; miR-125a-3p decreases alongside with an increase in Fyn expression. Microinjection of miR-125a-3p inhibits GVBD, an effect that is markedly reduced by Fyn over-expression, and impairs the organization of the actin rim surrounding the nucleus. Lower rate of GVBD is also observed in oocytes exposed to cytochalasin-D or blebbistatin, which interfere with actin polymerization and contractility of actin bundles, respectively. By down-regulating Fyn in HEK-293T cells, miR-125a-3p reduces the interaction between actin and A-type lamins, which constitute the nuclear-lamina. Our findings suggest a mechanism, by which a decrease in miR-125a-3p during oocyte maturation facilitates GVBD by allowing Fyn up-regulation and the resulting stabilization of the interaction between actin and A-type lamins.

## Introduction

Meiosis of mammalian oocytes begins during embryonic life when oogonia enter the first meiotic division (from then on referred to as oocytes) and arrest at the diplotene stage of the first prophase, characterized by the presence of a prominent nucleus, known as the germinal vesicle (GV). The development and differentiation of ovarian follicles is accompanied by growth of the resident GV oocytes. A surge of LH stimulates fully-grown GV oocytes, which reside within large preovulatory antral follicles, to resume meiosis and undergo maturation. This process is manifested by chromosomes condensation, GV breakdown (GVBD), spindle formation and its migration towards the oocyte cortex, segregation of homologous chromosomes, extrusion of the first polar body (PBI) and an arrest at metaphase of the second meiotic division (MII). A vast array of tightly-regulated signal transduction pathways control the oocyte meiotic arrest and enable maturation at a time- and space-restricted manner (reviewed in^1, 2^).

One of the signaling pathways that have been widely explored during oocyte maturation is that of the Src-family kinases (SFKs). Inhibition of SFKs by SU6656 or PP_2_ in GV mouse oocytes, decreased the rate of GVBD, exit from first meiotic division and PBI extrusion^3, 4^. This treatment also caused spindle structure-anomalies and increased PBI volume^3^. SFKs, whether phosphorylated on tyrosine 416 or not, enter the GV of mouse oocytes prior to GVBD^4^, supporting the concept that SFKs play a distinct role in GVBD. Inhibition of Fyn, an SFK which is highly expressed in oocytes^5^, significantly decreased the rate of GVBD^3^. Fyn is localized to actin filaments in both GV^6^ and MII oocytes^7^ and its activity is required to support cortical actin meshwork^7, 8^.

The resumption of meiosis in oocytes, also known as oocyte maturation, as well as early embryogenesis occur in the absence of transcription activity, utilizing pre-synthesized maternal mRNA transcripts, susceptible to post-transcriptional regulation^9, 10^. Several mechanisms of post-transcriptional regulation, including RNA interference by small interfering RNAs (siRNAs)^11^, were indicated in mouse oocytes (reviewed in^12^). Even though several studies suggest that maternal microRNAs (miRNAs) are relatively active in fully grown GV oocytes^13^ and regulate genes involved in oocyte maturation^14^, activation and early embryogenesis^15^, post-transcriptional regulation by maternal miRNAs remains controversial (reviewed in^16^).

We have recently found that miR-125a-3p post-transcriptionally regulates the expression of Fyn by a direct binding to Fyn 3′UTR, both in HEK 293 T cells^17^ and in granulosa cells^18^. Over-expression of miR-125a-3p in a prostate cancer cell line impaired the organization of actin filaments^19^.

The tight bond between Fyn and actin filaments, along with emerging role of actin filaments in nuclear envelope breakdown of large cells (reviewed in^20^) led us to assume that Fyn facilitates GVBD of mouse oocytes through regulating the organization and dynamics of actin filaments. We demonstrate that upon the ovulatory signal, the level of miR-125a-3p decreases, thus enabling translation of Fyn. Fyn is required for maintaining the interaction of GV-surrounding actin-filaments with A-type lamins prior to resumption of the first meiotic division; thus, hinting towards the mechanism by which miR-125a-3p, through regulating Fyn, enables GVBD of mouse oocytes.

## Results

### The expression profile of miR-125a-3p and Fyn during oocyte maturation

**Figure 1:**
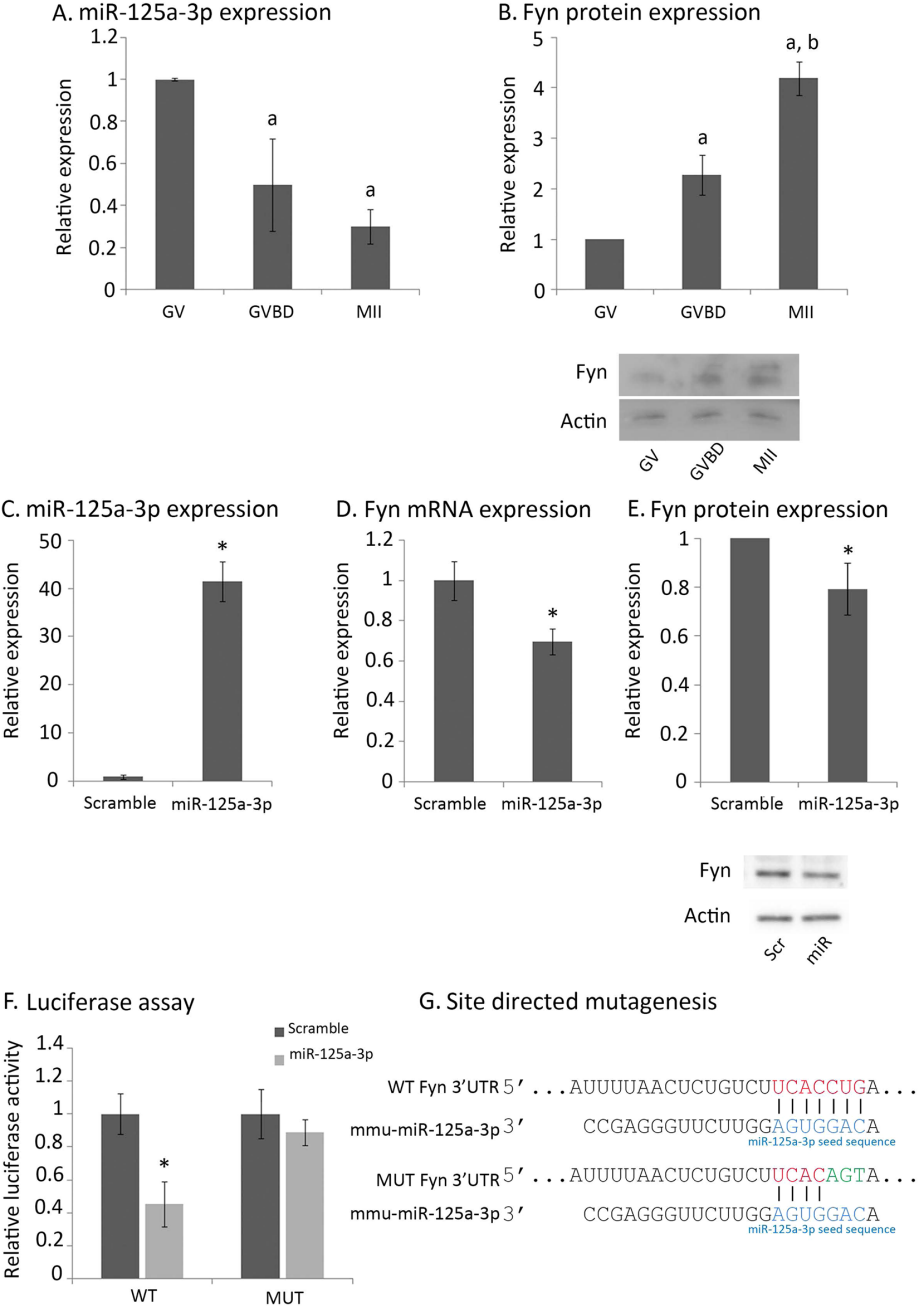
miR-125a-3p down-regulates Fyn expression in oocytes by a direct binding to Fyn 3′UTR. (**A**,**B**) Inverse correlation between miR-125a-3p and Fyn protein during maturation: RNA (30 oocytes at each experimental group) and protein (75 oocytes at each experimental group) were extracted from GV, GVBD and *in-vivo* matured MII oocytes. Whole cell lysates were subjected to (**A**) qPCR analysis for the detection of miR-125a-3p and U6-snRNA, which served as an internal control; or to (**B**) western blot analysis for detection of Fyn protein expression, with actin serving as an internal control (upper – graph; lower – a representative blot). Results were normalized to the value of GV oocytes. Data were analyzed by one-way ANOVA followed by Tukey post hoc test. Bars are Mean ± SD of four (**A**) or three (**B**) independent experiments. ^a,b^p < 0.05; ^a^versus GV oocytes; ^b^versus MII oocytes. (**C**–**E**) GV oocytes were microinjected with miR-125a-3p mimic (miR-125a-3p) or scramble-miR (Scramble) as control, and incubated for four hours at 37 °C in the presence of 1 µM milrinone. RNA (30 oocytes at each experimental group) and proteins (75 oocytes at each experimental group) were extracted. Whole cell lysates were subjected to qPCR analysis for detection of (**C**) miR-125a-3p and U6-snRNA, which served as an internal control, or of (**D**) Fyn mRNA; or subjected to western blot analysis for detection of (**E**) Fyn protein expression, with actin serving as an internal control (upper – graph; lower – a representative blot: Scramble-miR – Scr; miR-125a-3p - miR). Results were normalized to the value of scramble-miR. Data were analyzed by one-sample two-tailed student t-test. Bars are Mean ± SD of three independent experiments, *p < 0.05. (**F**) GV oocytes were microinjected with miR-125a-3p mimic (miR-125a-3p) or scramble-miR (Scramble) combined with a *Renilla*-luciferase RNA conjugated to either WT Fyn-3′UTR (WT) or to mutated Fyn-3′UTR (MUT). Oocytes were incubated for 4 hours at 37 °C in the presence of milrinone, prior to lysis. The results of each experimental group were normalized to the value of scramble-miR. Data were analyzed by one-way ANOVA followed by Tukey post hoc test. Bars are Mean ± SD of three independent experiments, *p < 0.05. (**G**) A scheme showing the binding-site of miR-125a-3p within WT Fyn 3′UTR of mouse origin, and the site-directed mutagenesis employed to prevent miR-125a-3p from binding to Fyn 3′UTR.

To characterize the expression profile of miR-125a-3p and Fyn throughout oocyte maturation, mouse oocytes, at various stages of meiosis (GV, GVBD and MII) were lysed and their RNAs and proteins were extracted and subjected to qPCR and WB, respectively. We found the patterns of miR-125a-3p and Fyn expression to be reciprocal: whereas the expression level of miR-125a-3p decreased during maturation, that of Fyn increased (Fig. 1A,B). The expression of miR-125a-3p decreased significantly already at the GVBD stage (p = 0.002) and continued to decrease through the MII stage. The expression of Fyn significantly increased during the transition from GV to GVBD (p = 0.001) and was followed by another significant increase at the MII stage (p = 0.001). These findings may suggest that Fyn, previously shown by us to participate in the control of oocyte maturation^3^, is post-transcriptionally regulated by miR-125a-3p in mouse fully grown GV oocytes.

### miR-125a-3p post-transcriptionally regulates Fyn expression in mouse oocytes

Because the ability of miRNAs in general, and of miR-125 family in particular, to regulate target genes is specific to cell type and to the differentiation stage^21^, we validated that miR-125a-3p can down-regulate the expression of Fyn in oocytes by microinjecting GV oocytes with miR-125a-3p mimic. We showed a 40-fold increase in miR-125a-3p expression, compared to oocytes microinjected with scramble-miR (Fig. 1C; p = 0.0002). Using qPCR analysis, we confirmed that Fyn mRNA is down-regulated in miR-125a-3p-over-expressing oocytes (Fig. 1D; p = 0.02). Over-expression of miR-125a-3p also caused a decrease in the level of Fyn protein expression (Fig. 1E; p = 0.04), though to a lesser extent than the decrease in Fyn mRNA. To determine whether miR-125a-3p down-regulates Fyn by a direct binding to its 3′UTR, we conducted a luciferase assay using an RNA construct that encodes Renilia-luciferase under the control of Fyn 3′UTR (WT). The specificity of the binding between miR-125a-3p and Fyn mRNA was examined using another RNA construct in which miR-125a-3p binding site within Fyn 3′UTR was mutated (MUT; illustrated in Fig. 1G). As expected, miR-125a-3p inhibited the expression of Renilla-luciferase under the control of WT Fyn 3′UTR, but it failed to inhibit the expression of Renilla-luciferase that was under the regulation of MUT Fyn 3′UTR (Fig. 1F; p = 0.001). Thus, these findings indicate that the post-transcription regulation of Fyn by miR-125a-3p in mouse GV oocytes is a result of a direct binding of miR-125a-3p to Fyn 3′UTR.

### miR-125a-3p inhibits GVBD in a Fyn-dependent manner

**Figure 2:**
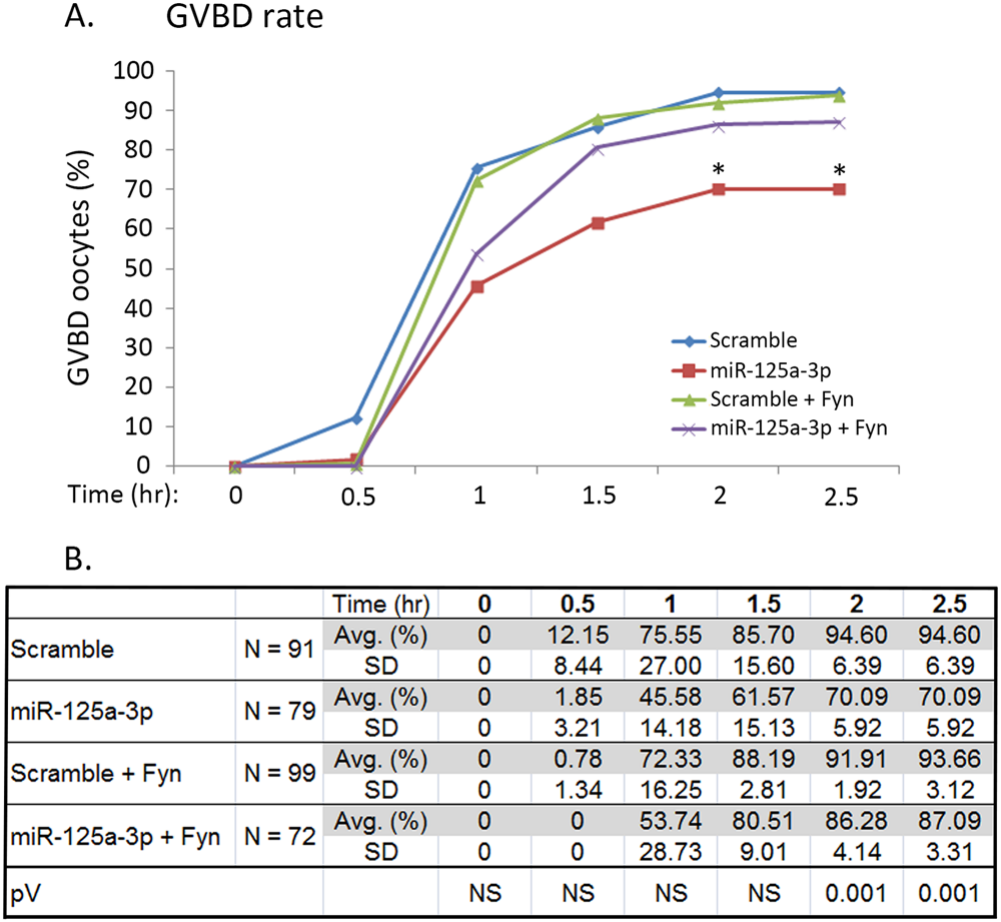
Over-expression of miR-125a-3p inhibits the rate of GVBD in a Fyn-dependent manner. GV oocytes were microinjected with either miR-125a-3p mimic (miR-125a-3p) or scramble-miR (Scramble), both either with or without Fyn RNA that lacks the 3′UTR region (miR-125a-3p + Fyn or, Scramble + Fyn). Oocytes were incubated for 4 hours at 37 °C in the presence of 1 µM milrinone, washed and transferred to fresh milrinone-free culture medium. Oocytes were monitored for GVBD every 30 minutes for 2.5 hours. The experiment was repeated three times. Results were analyzed by repeated measures ANOVA followed by Tukey post hoc test, and are presented in a line-chart, *p < 0.05 (**A**). The table (**B**) indicates the average of percent GVBD oocytes and SD of each dot presented at the line-chart.

We have previously described that Fyn plays a prominent role in oocyte maturation and that GVBD was inhibited in oocytes microinjected with a dominant-negative (DN) form of Fyn, or by exposure to an extracellular chemical inhibitor of this kinase^3^. Thus, we hypothesized that the decrease in miR-125a-3p expression during the GV-GVBD transition, which enables Fyn up-regulation, is a prerequisite to GVBD. To test this hypothesis we monitored both occurrence and timing of GVBD in miR-125a-3p-over-expressing oocytes. Microinjected GV oocytes were removed from culture medium supplemented with milrinone (to prevent spontaneous resumption of the first meiosis) and synchronously allowed to mature *in-vitro*. The presence of GV was recorded every 30 minutes for 2.5 hours, a time at which most oocytes undergo GVBD *in-vivo* ^22^ as well as *in-vitro* ^3^. The microinjection procedure did not hamper GVBD as ~95% of scramble-microinjected oocytes underwent GVBD 2 hours later, resembling GVBD rate of non-injected oocytes^3^. However, the rate of GVBD of miR-125a-3p over-expressing oocytes was significantly lower, 2 and 2.5 hours after microinjection, than that of scramble-microinjected oocytes (p = 0.001, at both time points), though miR-125a-3p had no effect on the timing of GVBD (Fig. 2). To ascertain that the effect of miR-125a-3p on GVBD is mediated by down-regulation of Fyn, we microinjected GV oocytes with scramble-miR or miR-125a-3p mimic together with an RNA construct encoding for WT Fyn, lacking Fyn 3′UTR sequence. The rate of GVBD in oocytes injected with miR-125a-3p and WT Fyn resembled that of oocytes microinjected with scramble-miR either with or without WT Fyn (Fig. 2). These findings suggest that miR-125a-3p exerts its inhibitory effect on GVBD by down-regulating Fyn expression. As in the case of miR-125a-3p, microinjection of WT Fyn had no effect on the timing of GVBD (Fig. 2).

### The GV of mouse oocytes is surrounded by an actin rim

**Figure 3:**
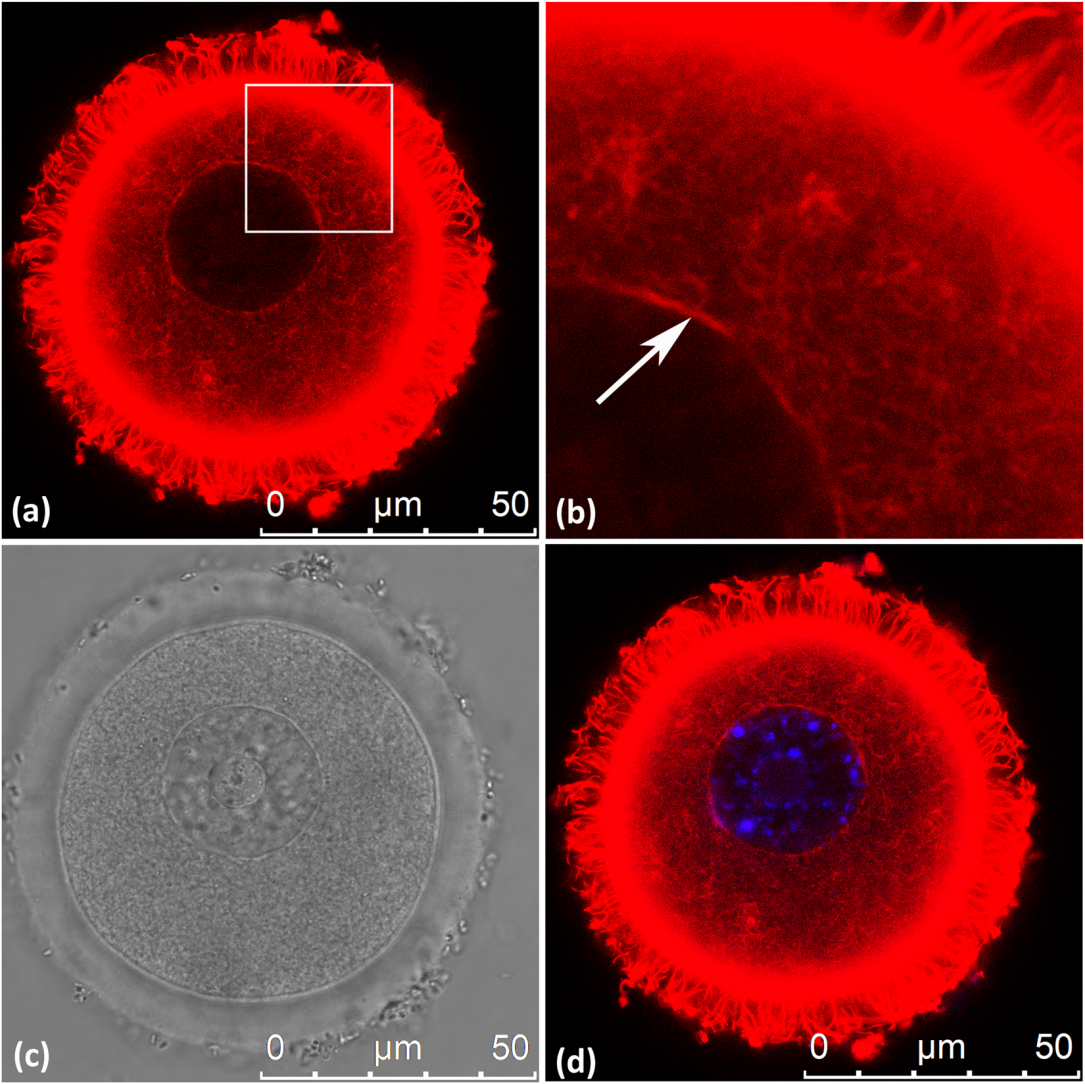
Nuclear actin rim surrounds the GV of mouse oocytes. GV oocytes were fixed and processed as described in ‘Experimental Procedures’. Actin filaments were stained with Tritc-Phalloidin (red) diluted in PBS, containing 0.1% Triton X-100 and 3% BSA. DNA was stained with Hoecst (blue). (**a**) Tritc-Phalloidin; (**b**) Enlargement of the area marked in (a); (**c**) Bright field; (**d**) Merge of Tritc-Phalloidin and Hoecst. White arrow points at the actin rim.

We have recently found that organization of actin cytoskeleton is impaired by miR-125a-3p in prostate cancer cells (Ninio-Many *et al*.^14^). Furthermore, Fyn is a known modulator of the actin cytoskeleton in several cell types^23, 24^. In mouse oocytes, Fyn colocalizes with actin microfilaments^6^ and its activity is required for the maintenance of cortical actin filaments network^8^. Thus, our explorations of the mechanism, by which miR-125a-3p inhibits GVBD through regulating Fyn, focused on a possible influence of miR-125a-3p and Fyn on the actin cytoskeleton. The presence of actin filaments in the nucleus is still controversial though a major progress has been made in support of this phenomenon (reviewed in^25, 26^). We first aimed at establishing the presence of actin filaments in the nuclei of mice oocytes. We found that the GV of non-treated mouse oocytes, fixed and stained with Tritc-Phalloidin, is surrounded by an actin rim (Fig. 3). Aside from this rim, we were unable to detect with Phalloidin any other structure of actin filaments within the nucleus.

### Organization of the nuclear actin rim is hampered in miR-125a-3p-microinjected oocytes

**Figure 4:**
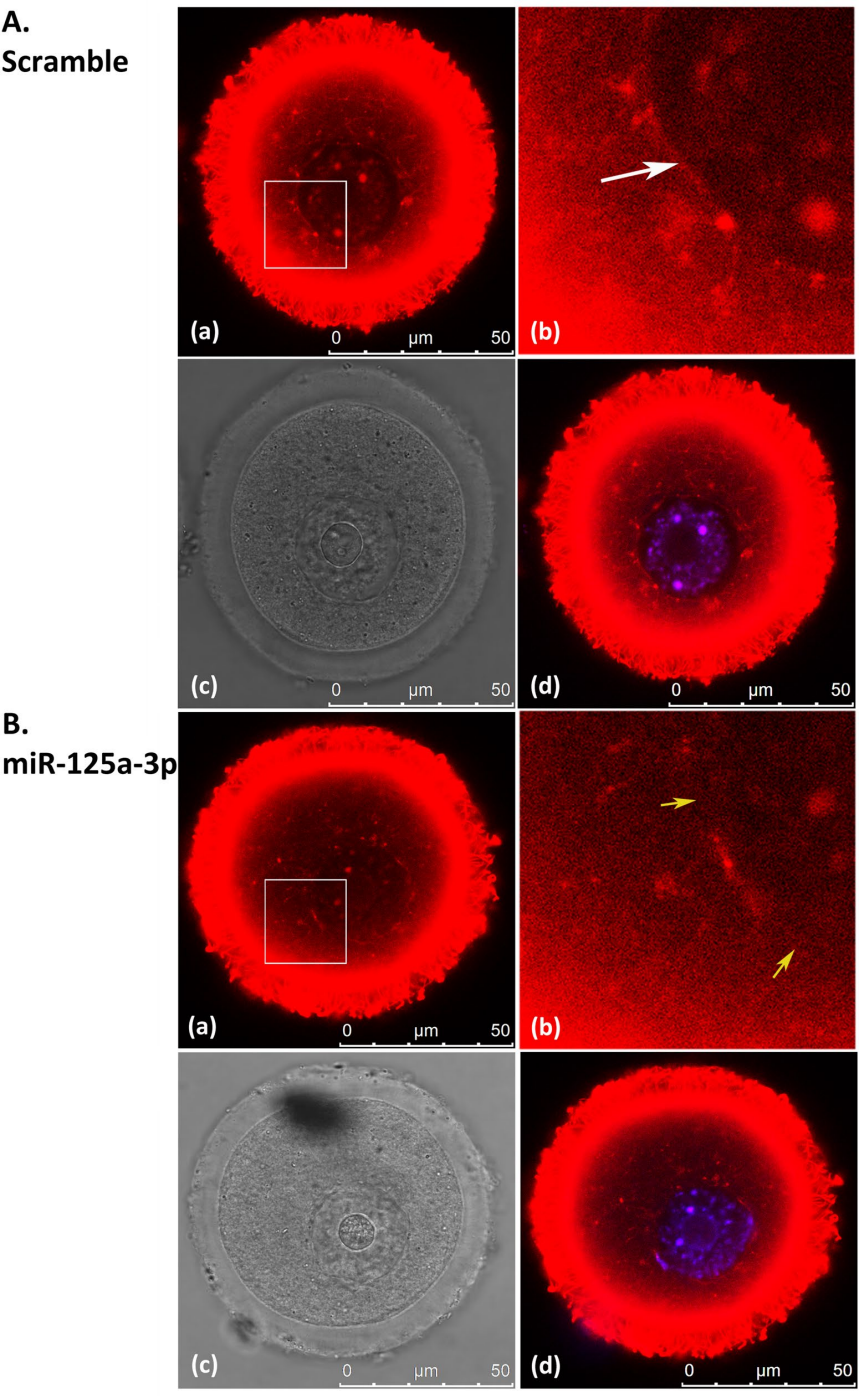
Over-expression of miR-125a-3p hampers the organization of the nuclear actin rim. GV oocytes were microinjected with either scramble-miR (**A**) or miR-125a-3p mimic (**B**). Oocytes were incubated for 4 hours at 37 °C in the presence of 1 µM milrinone prior to fixation performed as described in ‘Experimental Procedures’. Actin filaments were stained with Tritc-Phalloidin (red) diluted in PBS containing 0.1% Triton X-100 and 3% BSA. DNA was stained with Hoecst (blue). (a) Tritc-Phalloidin; (b) Enlargement of the area marked in (a); (c) Bright field; (d) Merge of Tritc-Phalloidin and Hoecst. White arrow points at the actin rim of a scramble-microinjected oocyte. Yellow arrows point at gaps within the actin rim of a miR-125a-3p-microinjected oocyte.

We microinjected GV oocytes with either miR-125a-3p mimic or scramble-miR to examine whether over-expression of miR-125a-3p in GV oocytes affects the organization of the nuclear actin rim. Microinjected oocytes were incubated for 4 hours, fixed and stained with Tritc-Phalloidin. The nuclear actin rim in oocytes microinjected with scramble-miR was intact and easily detected (Fig. 4A), whereas, the organization of the actin rim in miR-125a-3p-over-expressing oocytes was hampered; it was incomplete, but did not disappear entirely (Fig. 4B). Over-expression of miR-125a-3p seemed to have a negative effect on cytoplasmic actin filaments as well. Taken together, these findings suggest that miR-125a-3p modulates the organization of actin filaments in GV oocytes, including the nuclear actin rim.

### Actin filaments are involved in GVBD of mouse oocytes

**Figure 5:**
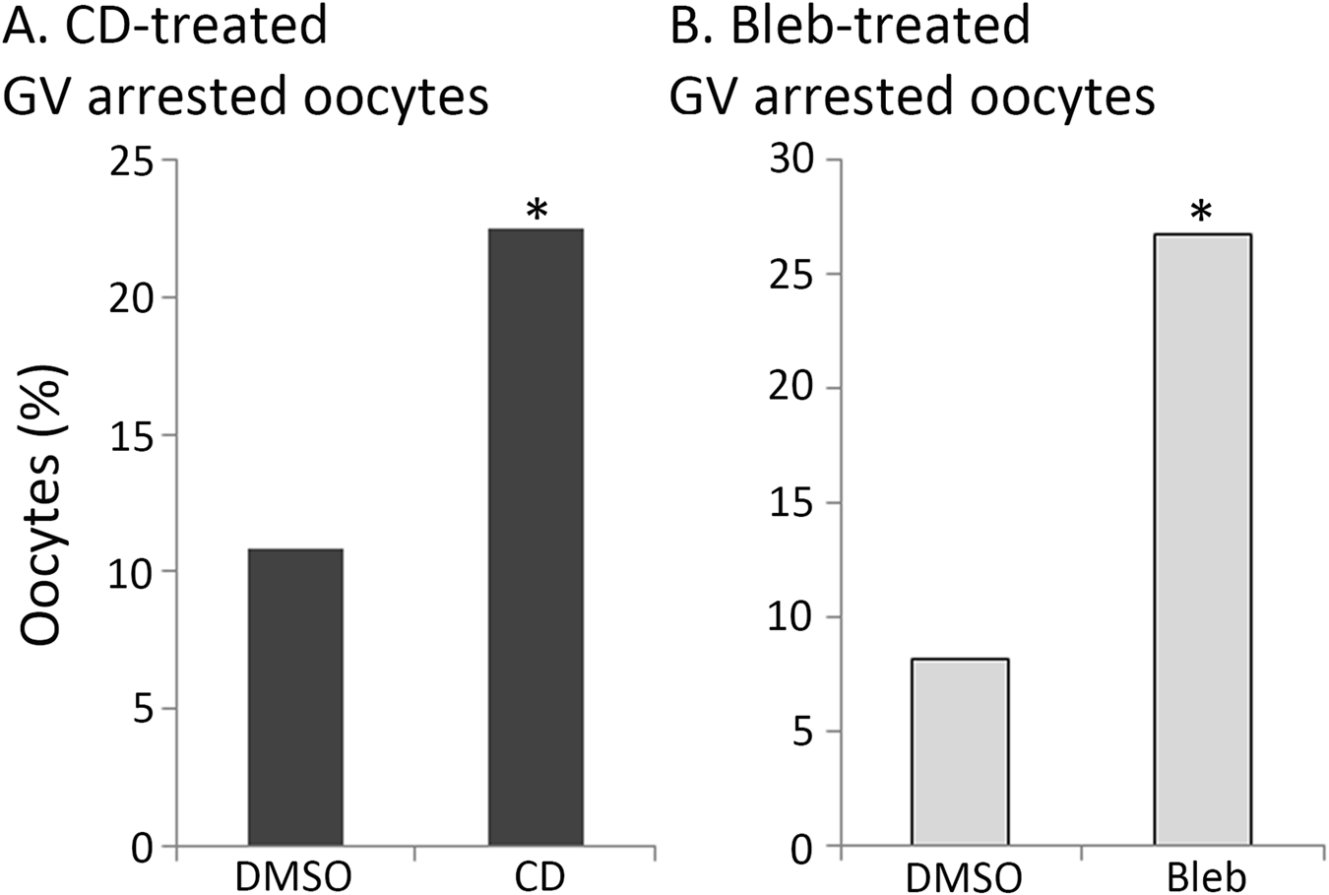
Polymerization and contractility of actin filaments support GVBD. GV oocytes were incubated for one hour at 37 °C at an atmosphere of 5% CO_2_ in the presence of 1 µM milrinone and 5 µg/ml cytochalasin D (CD; **A**), 50 µM blebbistatin (Bleb; **B**) or of an equal volume of vehicle (DMSO). Oocytes were washed out of milrinone and incubated for 3 more hours, to allow GVBD, in the presence of the inhibitors. The percentage of GV-arrested oocytes after 3 hours of incubation is presented. Results were analyzed by Chi-square test, χ^2^ = 0.006, for CD-treated oocytes; χ^2^ = 0.0003, for blebbistatin-treated oocytes.

The inhibitory effect of miR-125a-3p on GVBD and on the organization of the actin rim can be attributed either to a single pathway, which affects both, or to two distinct, independent mechanisms of action downstream to miR-125a-3p. In most species, actin filaments participate in resumption of the first meiotic division mainly through regulating spindle migration, positioning and anchorage, cortex polarity, chromosomes separation and cytokinesis (reviewed by ref. 27), whereas the involvement of actin filaments in GVBD is controversial. Yet, the presence of an actin rim surrounding the GV implies the involvement of actin filaments in maintaining the integrity of the GV and possibly mediating nuclear events such as GVBD. To challenge this option we exposed GV oocytes to cytochalasin D (CD), a potent inhibitor of actin polymerization, in a milrinone-containing culture medium. Following the pre-incubation period, oocytes were transferred into a milrinone-free medium and allowed to undergo GVBD. Monitoring GVBD we found a twofold increase in the number of CD-treated oocytes that failed to resume the first meiotic division than in DMSO-treated oocytes; exhibiting a prominent GV even after 4 hours of incubation in milrinone-free medium (Fig. 5A; χ^2^ = 0.006). To verify the role of actin filaments in GVBD of mouse oocytes, we performed the same experiment though using blebbistatin, a myosin II inhibitor that prevents the assembly of contractile actin bundles. Blebbistatin also decreased the rate of GVBD, as evidenced by more than threefold increase in the number of oocytes exhibiting a prominent GV (Fig. 5B; χ^2^ = 0.0003). However, it should be noted that in both groups the majority of the oocytes resumed meiosis and have undergone GVBD (77% and 73% of CD- and blebbistatin-treated oocytes, respectively). These findings imply that actin filaments play a role in GVBD of mouse oocytes.

### miR-125a-3p interferes with the interaction of actin with A-type lamins

**Figure 6:**
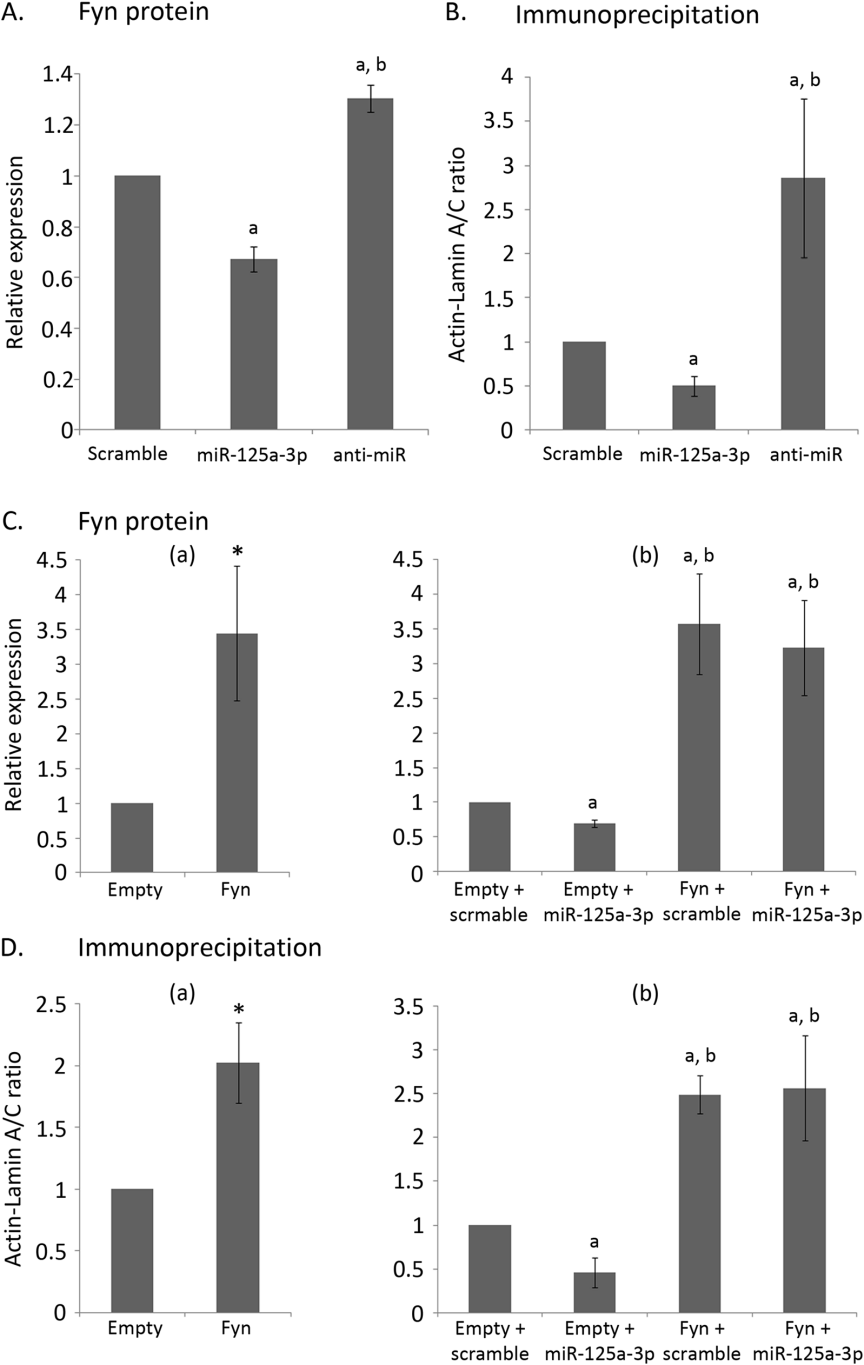
miR-125a-3p decreases the interaction of A-type lamins and actin in a Fyn-dependent manner. (**A**,**B**) HEK-293 T cells, transfected with either miR-125a-3p mimic (miR-125a-3p), anti-miR or scramble-miR (Scramble), were incubated for 24 hours, lysed and subjected to (**A**) WB analysis using anti-Fyn antibody and anti-actin as a loading control, or to (**B**) immunoprecipitation with anti-lamin A/C antibody and immunoblotted with anti-actin and anti-lamin A/C. Results are normalized to scramble. Data were analyzed by one-way ANOVA followed by Tukey post hoc test. Bars are Mean ± SEM of at least three independent experiments. ^a,b^p < 0.05; ^a^versus Scramble; ^b^versus miR-125a-3p. (**C**,**D**) HEK-293 T cells were transfected with Fyn-encoding vector (lacking the 3′UTR region; Fyn) or empty vector (Empty) serving as control (a); or transfected with either Fyn-encoding vector or empty vector together with either miR-125a-3p mimic (Empty + miR-125a-3p; Fyn + miR-125a-3p) or scramble-miR (Empty + scramble; Fyn + scramble) (b). Transfected cells were incubated for 24 hours, lysed and subjected to (**C**) WB analysis using anti-Fyn antibody and anti-actin as loading control, or to (**D**) immunoprecipitation with anti-lamin A/C antibody and immunoblotted with anti-actin and anti-lamin A/C. (a) Results are normalized to Empty. Data were analyzed by one-sample two-tailed student t-test. Bars are Mean ± SEM of five independent experiments, *p < 0.05. (b) Results are normalized to Empty + scramble. Data were analyzed by one-way ANOVA followed by Tukey post hoc test. Bars are Mean ± SEM of at least three independent experiments. ^a, b^p < 0.05; ^a^versus Empty + scramble; ^b^versus Empty + miR-125a-3p.

Actin filaments can directly bind A-type lamins, which constitute the nuclear lamina^28^, and indirectly through nuclear envelope proteins^29^. We hypothesized that the depolymerization of cytoplasmic actin filaments and of nuclear actin rim, observed in miR-125a-3p over-expressing GV oocytes, interferes with the interaction between actin and A-type lamins and may account for the inhibitory effect of miR-125a-3p on GVBD. To examine this hypothesis we used HEK 293 T cells transfected with miR-125a-3p mimic, anti-miR or scramble-miR as control. We initially validated that the expression of Fyn protein is decreased in miR-125a-3p over-expressing cells and increased in cells treated with anti-miR (Fig. 6A; p = 0.001 in both cases) followed by an immunoprecipitation (IP) assay using lamin A/C antibody. When exposing the immunoprecipitated complexes to actin antibody we found a dramatic decrease in the fraction of actin bound to A-type lamins in miR-125a-3p-over-expressing cells, as compared to cells transfected with scramble-miR (p = 0.005). Furthermore more actin was bound to A-type lamins in cells treated with anti-miR (Fig. 6B; p = 0.006).

### Inhibition of actin and A-type lamins interaction by miR-125a-3p is mediated through Fyn

As miR-125a-3p can potentially target several genes, we examined whether this effect of miR-125a-3p is mediated through its ability to inhibit the expression of Fyn. For this purpose, we over-expressed Fyn in HEK 293 T cells (Fig. 6C; (a); p = 0.02) and evaluated the relative level of bound actin. Over-expression of Fyn caused a twofold increase in the amount of actin bound to A-type lamins (Fig. 6D; (a); p = 0.01), similar to the results obtained using anti-miR. Next, cells were co-transfected with Fyn encoding vector lacking Fyn 3′UTR or an empty vector as control, combined with either scramble-miR or miR-125a-3p mimic. Over-expression of Fyn in transfected cells was confirmed by examining the level of Fyn protein expression. The level of Fyn expression in cells co-transfected with Fyn vector and miR-125a-3p mimic was similar to the level in cells co-transfected with Fyn vector and scramble-miR, indicating that over-expression of Fyn, caused by Fyn-encoding vector that lacks Fyn 3′UTR, overcomes the inhibitory effect of miR-125a-3p on the endogenous Fyn (Fig. 6C; (b); p = 0.024 and p = 0.009, versus cells co-transfected with empty vector and scramble-miR, respectively). We evaluated the interaction between actin and A-type lamins in co-transfected cells subjected to IP. In cells co-transfected with Fyn vector and miR-125a-3p, not only was the miR-125a-3p-mediated decrease in the amount of actin bound to A-type lamins prevented, but the over-expression of Fyn caused an increased interaction of actin and A-type lamins bringing it to the level found in cells co-transfected with Fyn and scramble-miR (Fig. 6D; (b); p = 0.012 and p = 0.008, versus cells co-transfected with empty vector and scramble-miR, respectively). These findings indicate that the miR-125a-3p-mediated decrease in actin and A-type lamins interaction is Fyn dependent.

### Fyn does not form a complex with actin and A-type lamins

**Figure 7:**
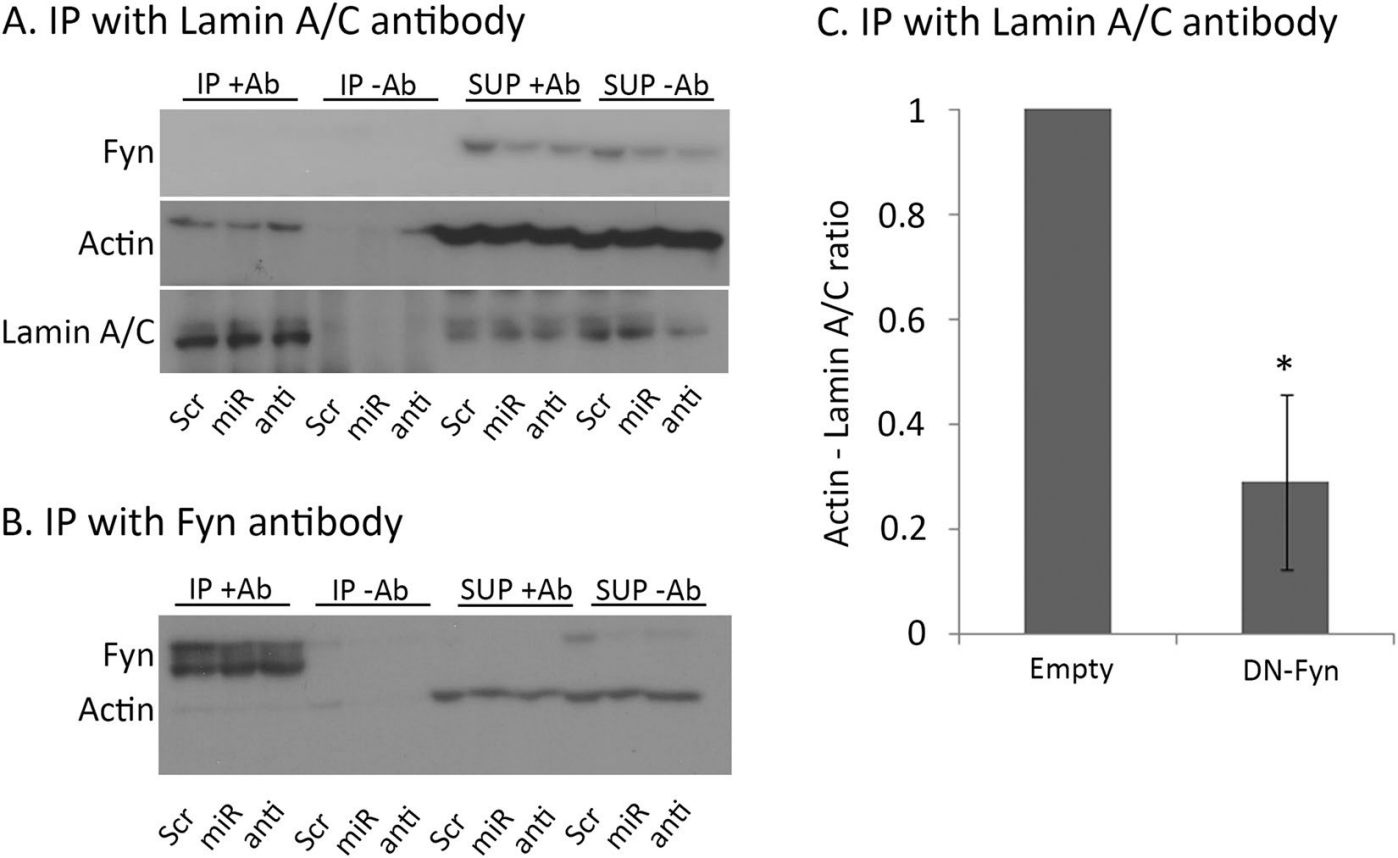
Fyn does not generate a complex with either A-type lamins or actin, but its activity is required for stabilization of the interaction of A-type lamins and actin. (**A**,**B**) HEK-293 T cells were transfected with either miR-125a-3p mimic (miR), anti-miR (anti) or scramble-miR (Scr), incubated for 24 hours, lysed and immunoprecipitated (IP + Ab) with (**A**) anti-lamin A/C antibody. The immunoprecipitated complexes were immunoblotted with anti-Fyn, anti-actin and anti-lamin A/C antibodies or immunoprecipitated with (**B**) anti-Fyn antibody and then immunoblotted with anti-actin and anti-Fyn antibodies. A negative control with no added antibody (IP-Ab) is displayed for each treatment. The supernatant that remained after immunoprecipitation is also displayed (SUP + Ab, SUP-Ab). (**C**) HEK-293 T cells were transfected with an empty-vector (Empty) or a vector encoding for dominant-negative form of Fyn (DN-Fyn), incubated for 24 hours, lysed and immunoprecipitated with anti-lamin A/C antibody and then immunoblotted with anti-actin and anti-lamin A/C antibodies. Data were analyzed by one-sample two-tailed student t-test. Bars are Mean ± SEM of three independent experiments, *p < 0.05.

As a member of the Src-Family Kinases, Fyn is known for its ability to form protein-protein interactions^30^. To test whether Fyn is part of the complex of actin and A-type lamins, HEK 293 T cells were transfected with miR-125a-3p mimic, anti-miR or scramble-miR as control, lysed and subjected to IP assay using the lamin A/C antibody. No Fyn was detected in the immunoprecipitated complexes of all treated cells; on the contrary, Fyn was easily detected in the supernatant left after the proteins were immunoprecipitated (Fig. 7A). To eliminate the possibility of a technical problem we exposed the immunoprecipitated complexes to actin antibody and found, as expected, a decreased level of actin in miR-125a-3p-over-expressing cells and an increased level of this protein in cells treated with anti-miR (Fig. 7A).

Fyn also interacts with several cytoskeletal proteins^24^ and co-localizes with actin in GV and MII mouse oocytes^6^. This observation led us to examine whether Fyn is bound to actin filaments with no dependency on A-type lamins. We repeated the experiment of immunoprecipitating the complex of proteins, using this time the Fyn antibody. We were unable to detect actin in the Fyn-immunoprecipitated complexes, though it was easily detected in the supernatant (Fig. 7B). Thus, we assume that Fyn is not found in a strong interaction with either A-type lamins or actin.

### Fyn activity is necessary for stabilizing the actin and A-type lamins interaction

After showing that Fyn is not an integral part of the actin and A-type lamins complex, the question whether the activity of Fyn is necessary for stabilizing actin and A-type lamins interaction, remains open. To address this issue HEK 293 T cells were transfected with either an empty vector or a vector that contains a dominant-negative form of Fyn (DN-Fyn). Similar to the previous experiment, complexes were immunoprecipitated with lamin A/C antibody. Exposing the complexes to anti-actin antibody we found a dramatic decrease in the amount of actin bound to A-type lamins (Fig. 7C; p = 0.035), inferring that the Fyn activity is required for maintaining a strong interaction between actin and A-type lamins.

## Discussion

Fyn, which is localized to the oocytes cortex, the spindle pole and cleavage furrow^3^, has been previously investigated in mammalian oocytes and its seminal role in oocyte maturation is well established. Fyn-null female mice are sub-fertile; they produce a similar number of oocytes as wild-type females, however a high proportion of them fails to mature with many of the mature oocytes displaying spindle and chromosomes abnormalities^31^. Oocytes of Fyn-null mice also show alterations in the tyrosine phosphorylation pattern^32^ as well as in the distribution of microvilli and cortical granules^8^. Inhibition of Fyn by microinjection of a DN form of this kinase resulted in a decrease in GVBD, MI exit and first polar body extrusion^3^.

We have previously shown that miR-125a-3p regulates Fyn post-transcriptionally in HEK-293T cells^17^ and in rodents’ ovarian granulosa cells^18^. We further demonstrated that this effect is executed by a direct binding to Fyn 3′UTR. The expression of miR-125a-3p, which decreases in mural granulosa cells exposed to hCG *in-vivo* and *in-vitro*, enables up-regulation of Fyn. Interfering with this regulatory pathway *in-vivo* reduced the number of ovulated oocytes^18^. Herein we show a similar reciprocity of miR-125a-3p and Fyn expression patterns in oocytes towards ovulation. Upon resumption of the first meiotic division, during the transition from GV to GVBD, the level of miR-125a-3p decreases while that of Fyn protein increases. Over-expression of miR-125a-3p in GV oocytes resulted in a decrease in Fyn mRNA and protein levels. Luciferase assay confirmed that similar to the granulosa cells, miR-125a-3p down-regulates the expression of Fyn in oocytes by a direct binding to Fyn 3′UTR. Currently, we cannot rule out the possibility that either RNA degradation or translation inhibition are at the core of the decrease in Fyn protein expression imposed by over-expression of miR-125a-3p.

We further showed that GVBD was inhibited by 30% in GV oocytes microinjected with miR-125a-3p mimic. To validate that miR-125a-3p exerts its inhibitory effect on GVBD through Fyn down-regulation, we microinjected oocytes with WT Fyn RNA that lacks the Fyn 3′UTR sequence, together with either miR-125a-3p mimic or scramble-miR. The fraction of oocytes undergoing GVBD following microinjection of scramble-miR was similar, when injected with or without Fyn, (93.7% or 94.6%, respectively); indicating that Fyn does not affect GVBD when the level of miR-125a-3p is physiological. However, when Fyn was microinjected together with miR-125a-3p mimic it moderated the inhibitory effect of miR-125a-3p, resulting in 87% GVBD. No significant differences in GVBD were found between control oocytes and oocytes microinjected with miR-125a-3p in combination with Fyn, implying that down-regulation of Fyn may be the main, though not the exclusive, mechanism by which miR-125a-3p inhibits GVBD.

The partial inhibition of GVBD in miR-125a-3p-over-expressing oocytes can be the result of a moderate decrease in Fyn protein expression achieved during the four hours of incubation following microinjection, and is directly influenced by the Fyn’s half-life. Though only a moderate decrease in Fyn protein expression was achieved, we may assume that de-novo synthesis of Fyn, which takes place during the GV to GVBD transition, is inhibited by over-expression of miR-125a-3p. Moreover, the rate of GVBD achieved in miR-125a-3p over-expressing oocytes was similar to that of oocytes microinjected with DN-Fyn^3^. Thus, these findings suggest that either Fyn is not the major contributor to mouse GVBD, or that a possible compensation by other SFKs reduces the inhibitory effect of miR-125a-3p over-expression on GVBD.

It is largely accepted that, similar to nuclear envelope breakdown (NEB) in somatic cells, phosphorylation of nuclear envelope proteins is an initial event in GVBD, which leads to dispersal of the nuclear pore complexes and disassembly of the nuclear lamina (reviewed by refs 33, 34). The first visible signs of nuclear lamina disassembly can be detected as early as at the prophase with the dissociation of A-type lamins and their release into the nucleoplasm. Changes in B-type lamins appear only after the dispersal of the nuclear pore complexes^35^. It is accepted that both phosphorylation of B- and A-type lamins^36, 37^ and microtubule-induced mechanical stretching of the lamina^38^ participate in nuclear lamina disassembly. However, several studies indicated that NEB occurs in cells with nocodazole-disrupted microtubules^39, 40^, though in a delayed fashion^41^. Furthermore, Panorchan *et al*. showed that the deformation induced by microtubules is of a lower magnitude than required to break the nuclear lamina network^42^. Hence, it is possible that other factors are involved in the process of nuclear lamina disassembly during NEB. Here, we show that GV in mouse oocytes is surrounded by an actin rim. Treatment with CD, a potent actin depolymerization agent, inhibits GVBD in mouse oocytes, as evidenced by the twofold increase in the number of oocytes that remained arrested at the GV stage. Blebbistatin, a myosin II inhibitor that prevents the assembly of contractile actin bundles, had a similar effect on GVBD, causing a 3.3-fold increase in the number of GV-arrested oocytes. Actin polymerization is required for remodeling of the perinuclear actin rim in murine fibroblasts, whereas blebbistatin has no effect on the assembly of the actin rim^43^. On the contrary, in cancerous epithelial cell lines, nuclear envelope rupture was inhibited by both CD and blebbistatin, which caused the loss of contractile actin bundles associated with the nucleus^44^. Because the majority of CD- and blebbistatin-treated oocytes resumed the first meiotic division, our results suggest that though not essential, polymerization and contractility of actin filaments contribute to GVBD in mouse oocytes.

We further showed that microinjection of miR-125a-3p, which inhibits GVBD, also hampered the organization of nuclear actin rim, forming large gaps in it, whereas the nuclear actin rim in scramble-microinjected oocytes was clearly visible. The rate of GV-arrested oocytes in miR-125a-3p-over-expressing oocytes was of the same magnitude as in CD- or blebbistatin-treated oocytes; suggesting that the inhibitory effect of miR-125a-3p on GVBD may be attributed to the damage caused to the perinuclear actin filaments.

Actin filaments surrounding the nuclei of mouse oocytes were also described by Azoury *et al*. who showed that the changes in density and dynamics of actin filaments were concomitant with the increase in nuclear permeability just before GVBD^45^. Luo *et al*. showed that depletion of SUN1 or KASH5, which constitute the linker of nucleoskeleton and cytoskeleton (LINC) complex localized on the nuclear envelope, led to GV-arrest of mouse oocytes. Oocytes that underwent GVBD exhibited abnormal spindle and decrease in the intensity of cytoplasmic actin filaments labeling^46^. In Xenopus oocytes, actin filaments were found in the nucleus^47, 48^ and stabilization of actin filaments by microinjection of phalloidin prevented GVBD and altered the organization of microtubules^49^. In starfish oocytes, in which the process of GVBD is not dependent on microtubules^50^, a mesh of actin filaments polymerizes transiently on the inner nuclear membrane and drives GVBD^51^. Thus, our findings are in agreements with recent studies suggesting that actin filaments facilitate NEB/GVBD in large cells, where microtubules cannot bridge the whole nucleus^20^, though actin filaments seem to support NEB also in somatic cells^52^.

Interaction of actin filaments with A-type lamins might be at the core of the mechanism by which actin filaments support NEB/GVBD. Simon *et al*. described a direct binding of actin filaments with A-type lamins^28^. A-type lamins (A, C, AΔ10 and C2) are derived from alternative splicing of a single mammalian gene (reviewed by ref. 53). Both A-type and B-type lamins possess a conserved actin binding site and bind actin filaments *in-vitro* directly; however, A-type lamin has another unique actin binding site in its carboxy terminal domain, which may explain the stronger interaction of actin filaments with lamin A than with lamin B or lamin C^28^. Direct binding of actin filaments and A-type lamins would require the presence of actin in the nucleus. Though nuclear actin has long been debated, ample evidence support the presence of actin in the nucleus and its importance for nuclear processes such as transcription and chromatin remodeling (reviewed by refs 26, 54 and 55). Furthermore, many actin-binding proteins have been identified in the nucleus (for review of actin-binding proteins found in the nucleus see refs 54, 56). We were unable to detect other structures of nuclear actin filaments aside for the actin rim; but lack of staining is not sufficient for concluding that the GV of mouse oocytes is devoid of actin filaments, as binding of short actin filaments to actin-binding proteins may hinder the binding of phalloidin^57^.

An indirect interaction of actin filaments and A-type lamins through actin- and lamin-binding proteins has also been described (reviewed by ref. 55). Some actin-binding proteins are localized to the nuclear envelope, connecting the nucleoskeleton with the cytoskeleton^29, 58, 59^. One of these proteins, SUN1, which is part of the LINC complex and resides in the inner nuclear membrane, binds also A-type lamins^29^. Furthermore, a perinuclear actin rim connected to the LINC complex is present in somatic cells but absent from cells with A-type lamins deficiency^60^. Emerin, also localized to the inner nuclear membrane, binds both A-type and B-type lamins, as well as nuclear actin^61^, and is suggested to contribute to the formation of an actin cortical network at the nuclear inner membrane^62^.

To assess whether miR-125a-3p regulates the interaction between actin and A-type lamins we used lamin A/C antibody to immunoprecipitate proteins of HEK 293 T cells transfected with miR-125a-3p mimic, anti-miR-125a-3p or scramble-miR as control. The amount of actin bounds to A-type lamins in miR-125a-3p over-expressing cells was ~50% that of control scramble-transfected cells, whereas in cells transfected with anti-miR, more actin was bound to A-type lamins than in scramble-transfected cells. We confirmed that the level of Fyn was decreased in miR-125a-3p-transfected cells and increased in anti-miR-treated cells. Over-expression of Fyn resulted in a twofold increase in actin bound to A-type lamins, similar to the increase observed in anti-miR-treated cells. To verify that the decreased interaction between actin and A-type lamins in miR-125a-3p over-expressing cells is mediated by Fyn, we co-transfected cells with Fyn encoding plasmid that lacks the Fyn 3′UTR sequence, or an empty vector as control, together with either miR-125a-3p mimic or scramble-miR as control. When co-transfected with an empty vector, miR-125a-3p caused a decrease in both the expression of Fyn protein and in the amount of actin bound to A-type lamins. As expected, the decrease in Fyn expression mediated through over-expression of miR-125a-3p was prevented in cells co-transfected with miR-125a-3p and the Fyn encoding plasmid. Furthermore, over-expression of Fyn that is not subjected to the regulation of miR-125a-3p, completely blocked the miR-125a-3p-mediated decrease in the interaction of actin and A-type lamins. Thus, our findings suggest that Fyn supports the interaction of actin and A-type lamins and that over-expression of miR-125a-3p interferes with this interaction by inhibition of Fyn.

Along its phosphorylation activity, Fyn executes multiple protein-protein interactions^30, 63^, enticing us to look for the presence of Fyn in complex with actin and A-type lamins. We were not able to detect Fyn in lysates of all transfected cells after immunoprecipitation with lamin A/C antibody, nor were we able to detect actin in the lysates after immunoprecipitation with Fyn antibody. Thus, we concluded that Fyn does not interact strongly with either A-type lamins or actin, but did not exclude the possibility that the activity of Fyn is required to sustain the interaction of actin and A-type lamins. We transfected cells with a plasmid that encodes for a DN form of Fyn in which a point mutation has been made at L299 within Fyn ATP binding site, transforming Fyn to a kinase-dead mutant, capable of inhibiting endogenous Fyn^3^. The amount of actin bound to A-type lamins decreased by 70% in DN-Fyn over-expressing cells, inferring that the interaction of actin and A-type lamins necessitates the activity of Fyn. The decrease in actin and A-type lamins interaction in DN-Fyn over-expressing cells was more profound than that caused by over-expression of miR-125a-3p, probably due to the partial inhibition of Fyn in miR-125a-3p-over-expressing cells. Fyn may act directly or indirectly either on A-type lamins or on actin filaments. A direct effect of Fyn on A-type lamins would require the presence of Fyn in the nucleus, which was detected in somatic cells^64, 65^. However, in mouse oocytes Fyn was detected only in the GV of oocytes exhibiting a lower maturation competence^66^. Fyn co-localizes with and phosphorylates β-adducin, an actin filaments associated cytoskeletal protein^24^, supporting the possible action of Fyn on actin filaments. The mechanism by which Fyn promotes the interaction of actin and A-type lamins needs to be further explored.

In this study, we show a role for miR-125a-3p in mouse oocytes; miR-125a-3p participates in maintaining oocytes arrested at prophase I by post-transcriptional regulation of Fyn expression. Upon the ovulatory signal, the level of miR-125a-3p decreases, enabling translation of Fyn, required for adequate GVBD, which is facilitated by actin filaments. We further show that the GV in mouse oocytes is surrounded by an actin rim, the assembly of which is hampered by over-expression of miR-125a-3p. Based on our results, we propose a model in which Fyn activity is required for maintaining the interaction of actin filaments and A-type lamins prior to resumption of first meiotic division, thus supporting GVBD of mouse oocytes.

### Experimental procedures

#### Animals

ICR female mice (Envigo RMS Limited, Jerusalem, Israel) were housed in temperature and humidity controlled rooms at the animal facilities of the Sackler Faculty of Medicine, Tel Aviv University, under artificial illumination for 12 hours daily. This study was approved by the Institutional Animal Care and Use Committee of Tel-Aviv University and performed in according to its guidelines.

#### Oocytes collection and culture

GV oocytes were released from large antral follicles of ovaries dissected from naïve 8 weeks old female mice into pre-warmed (37 °C) M2 medium (M-7167; Sigma-Aldrich, St. Louis, MO, USA) supplemented with penicillin (100 IU/ml), streptomycin (100 mg/ml; Biological Industries, Beit Ha’emek, Israel) and milrinone (1 µM; M-4659; Sigma-Aldrich). By inhibiting phosphodiesterase III, milrinone prevents the intra-oocyte drop in cAMP that leads to spontaneous resumption of the first meiotic division. Oocytes were stripped off their cumulus cells, the number of GV oocytes was recorded and they were either collected immediately or washed out of milrinone and cultured in a humidified atmosphere of 5% CO_2_ in air at 37 °C to allow synchronized resumption of meiosis. After three hours of incubation only oocytes that reinitiated their meiotic division, indicated by the absence of the GV (GVBD) were collected and their number was recorded. Mature, ovulated MII oocytes were recovered from the oviductal ampullae into pre-warmed M2 medium (Sigma-Aldrich) supplemented with penicillin and streptomycin 14 hours after hCG (7 IU; Sigma-Aldrich) administration to pregnant mare serum gonadotropin (PMSG)-primed female mice (5 IU; Syncro Part; Sanofi, Paris, France). Cumulus cells were removed by a brief exposure to 400 IU/ml hyaluronidase (H-3631; Sigma-Aldrich).

#### Cloning

The full-length sequences of human cDNAs encoding the WT or DN forms of Fyn kinase (I.M.A.G.E. clone ID number 3613878, ResGen, Carlsbad, CA, USA), inserted into pCMV–Tag 4 A (Stratagene, Cedar Creek, TX, USA) using the BamHI and EcoRI restriction enzymes (New England Biolabs, Ipswich, MA, USA), had already been constructed in our laboratory^67^. Fyn 3′UTR (of mouse origin) was cloned into psiCHECK-2 vector (Promega, Madison, WI, USA) using NotI and XhoI restriction enzymes (Fermentas, Burlington, Ontario, Canada^17^). Mutagenesis was performed using the QuikChange Site-Directed Mutagenesis Kit (Stratagene) as previously described^18^: nucleotides 5–7 at the miR-125a-3p binding site within Fyn 3′UTR (CTG; equivalent to nucleotides 2–4 in the seed sequence of miR-125a-3p) were replaced by AGT nucleotides.

#### *In-vitro* transcription

All plasmids were linearized using HpaI restriction enzyme (New England Biolabs; for pCMV–Tag 4 A vector) or NotI-HF restriction enzyme (New England Biolabs; for psiCHECK-2 vector). Linear plasmids were loaded on a 1% agarose gel and purified using the Wizard® SV Gel and PCR Clean-Up System (Promega). *In vitro* transcribed RNA transcripts, based on the pCMV–Tag 4 A vector, were prepared using the T3 mMESSAGE mMACHINE kit (AM-1348; Ambion, Austin, TX, USA). The same kit was used for psiCHECK-2 based vectors, though with a small modification: the T3 RNA polymerase was replaced with T7 RNA polymerase (AM2082; Invitrogen, Carlsbad, CA, USA) adjusted to the same final concentration (4 U/µl). Transcripts, based on the psiCHECK-2 vector, were subjected to poly-A tailing using the poly(A) tailing kit (AM1350; Ambion). All transcripts were extracted by phenol:chlorophome, precipitated by isopropanol and eluted by nuclease-free water to a concentration of 500 ng/ml.

#### Oocytes microinjections

Ovaries of 24–26 days old female mice primed with PMSG (5 IU) 48 hours earlier were excised into L-15 tissue culture medium (L-1518; Sigma-Aldrich) supplemented with 5% FBS, penicillin, streptomycin and 1 µM milrinone. Oocytes were released from the large antral follicles and transferred into 10 µl droplets of the above tissue culture medium under paraffin oil (18512; Sigma-Aldrich). Oocytes, containing an intact GV, were placed under an inverted DIC microscope (Axiovert 35; Zeiss, Oberkochen, Germany), and microinjected using an InjectMan NI2 micromanipulator and a FemtoJet automated microinjector (Eppendorf, Hamburg, Germany). Micropipettes were pulled manually using an M-97 Flaming/Brown micropipette puller (Sutter Instruments, Novato, CA, USA). *In-vitro* transcribed long RNA sequences (Renilla-luciferase-Fyn-3′UTR and WT-Fyn) were used at a final concentration of 250 ng/µl (~2.5 × 10^11^ molecules/µl). Short RNA sequences (scramble-miR; 4464059; Ambion, and miR-125a-3p mimic; MC12378; Thermo-Fisher Scientific, MA, USA) were used at a final concentration of 20.8 µM (12.5 × 10^12^ molecules/µl; 50 times higher than that of long RNA sequences). All RNAs were diluted in PBS:diethylpyrocarbonate-treated water (Biological Industries). Microinjected oocytes were incubated for 4 hours at 37 °C in a humidified incubator in L-15 tissue culture medium supplemented with 5% FBS, penicillin, streptomycin and 1 µM milrinone to allow for changes in genes expression.

#### Luciferase assay

GV oocytes were microinjected with RNA encoding for Renilla-luciferase under the regulation of WT Fyn 3′UTR or Fyn 3′UTR in which the miR-125a-3p binding site was mutated, together with either scramble-miR (4464059) or miR-125a-3p mimic (MC12378). After four hours of incubation equal numbers of oocytes from each experimental group were lysed and Renilla luciferase activity was assessed using the Renilla luciferase assay system (E2810; Promega), according to the manufacturer’s instructions. Luminescence readings were acquired using a TD 20/20 luminometer (Turner Design Incorporated, Sunnyvale, CA, USA).

#### Actin filaments staining

Oocytes were fixed for 60 minutes at 37 °C in a mixture of 100 mM HEPES (pH 7; titrated with KOH), 50 mM EGTA (pH 7; titrated with KOH), 10 mM MgSO_4_, 2% formaldehyde (methanol free) and 0.2% Triton X-100, as previously described^68^. Oocytes were left overnight at 4 °C in PBS with 0.1% Triton X-100. To stain the actin filaments oocytes were incubated for 1 hour at room temperature in phalloidin conjugated to TRITC (P1951; Sigma-Aldrich), diluted 1:500 in PBS supplemented with 0.1% Triton X-100 and 3% BSA and Hoechst (33342; Sigma-Aldrich) for DNA labeling. Images were acquired by a Leica laser confocal microscope (SP8; Leica Microsystems, Wetzlar, Germany).

#### Culture and transfection of HEK 293 T cells

Cells were maintained in DMEM (Biological Industries), supplemented with 10% fetal bovine serum (Life Technologies; Carlsbad, CA, USA), 2 mM L-glutamine, penicillin, and streptomycin (Biological Industries). Cells were maintained in a humidified atmosphere of 5% CO_2_ in air at 37 °C and transfected in 6-wells plates (Nunc, Copenhagen, Denmark) using Lipofectamine 2000 transfection reagent and Opti-MEM medium (Thermo-Fisher Scientific), according to manufacturer’s instructions. Scramble-miR (4464059), miR-125a-3p mimic (MC12378) and anti-miR-125a-3p (anti-miR; MH12378; Thermo-Fisher Scientific) were transfected at a final concentration of 5 nM. DNA plasmids were used at a final concentration of 0.167 µg/ml (0.5 µg total). Cells were incubated for 24 hours before being subjected to subsequent analysis.

#### Western blot (WB) analysis

HEK 293 T cells and oocytes (75 oocytes in each experimental group: GV, GVBD, MII) were lysed in 20 mM HEPES buffer containing 137 mM NaCl, 0.002% IGEPAL CA-630 (18896, Sigma-Aldrich), 2 mM vanadate, 1 mM PMSF and a cocktail of protease inhibitors (cOmplete, 11 836 145 001; Roche, Basel, Switzerland). Lysates were cleared by centrifugation at 13,300 rpm and an appropriate volume of sample buffer was added. Samples were boiled, subjected to 10% sodium dodecyl sulfate polyacrylamide gel electrophoresis (SDS-PAGE; Bio-Rad, Israel), transferred to a nitrocellulose membrane (Whatman GmbH, Dassel, Germany), immunoblotted with either anti-Fyn (NB500-517; Novus Biologicals, Oakville, Canada) or anti-actin (MAB1501; EMD Millipore, MA, USA) primary antibodies and incubated with anti-mouse horseradish-peroxidase conjugated (115-035-166; Jackson ImmunoResearch Laboratories; PA, USA) secondary antibody. The intensity of the bands was analyzed by the Image J software.

#### RNA isolation, reverse transcription and real-time PCR (qPCR)

Oocytes (30 from each experimental group) collected in 5 µl nuclease-free water, were lysed using the lysis buffer supplied in the TaqMan MicroRNA Cells-to-C_T_ Kit (AB-4391848; Ambion) according to manufacturer’s instructions. Reverse transcription of miRNAs was performed using 5 µl of Cells-to-C_T_ sample lysates and specific primers for miR-125a-3p (assay ID 2199; Applied Biosystems; Foster City, CA, USA) and 6 snRNA (assay ID 001973; Applied Biosystems). Real-time PCR was performed using 1.33 µl of the RT product. For detection of Fyn mRNA, total RNA was reverse transcribed using 13.2 µl of the Cells-to-C_T_ sample lysates and the High-Capacity cDNA Reverse Transcription Kit (4368814; Applied Biosystems). Real-time PCR was performed using 2 µl of the RT product, the SYBR green reagent (Power SYBR Green PCR Master Mix; Applied Biosystems) and Fyn specific primers (forward: 5′- ACCTCCATCCCGAACTACAAC-3′; reverse: 5′-CATAAAGCGCCACAAACAGTG-3′). In both procedures, the ABI Prism 7900 Sequence PCR machine (Applied Biosystems) was used.

#### Immunoprecipitation (IP)

HEK 293 T cells were lysed for 10 minutes at 4 °C in 50 mM HEPES (pH 7.4; titrated with KOH), 150 mM NaCl, 1 mM MgCl_2_, 1% Triton X-100, 1 mM PMSF, supplemented with protease inhibitors cocktail. Lysates were cleared by a 15 minutes centrifugation at 13,300 rpm and 4 °C. To eliminate non-specific binding, aliquots of cleared supernatants, containing 500 µg protein each, were incubated in constant rotation with 15 µl of protein A/G PLUS-Agarose Beads (SC-2003; Santa Cruz Biotechnology, CA, USA) for one hour at 4 °C and centrifuged at 13,300 rpm for two minutes. Pellets were discarded. Antibodies (anti-lamin A/C; 1:50, ab-108922; Abcam, Cambridge, UK; or anti-Fyn; NB500-517; 1:50) were added to the supernatant for an overnight incubation. Protein A/G PLUS-Agarose Beads were added for three additional hours of incubation. Both incubations were at 4 °C in constant rotation. The mixture was cleared and washed 3 times by centrifugation. The supernatant of the first centrifugation was used to evaluate the efficiency of the immunoprecipitation. Pellets were washed with lysis buffer devoid of Triton X-100. Proteins were removed from the beads by boiling them for 10 minutes in sample buffer; they were then resolved by 10% SDS-PAGE and transferred to a nitrocellulose membrane. Membranes were subjected to WB analysis using anti-lamin A/C, anti-Fyn and anti-actin (MAB1501) antibodies, detected by horseradish-peroxidase conjugated anti-rabbit (111-035-144; Jackson ImmunoResearch Laboratories; for anti-lamin A/C) and anti-mouse (for anti-Fyn and anti-actin) secondary antibodies.

#### Statistical analysis

SPSS (SPSS Inc, USA) was used for all statistical analyses. Data are expressed as means ± SD, unless otherwise mentioned. Data normality was assessed using Kolmogorov-Smirnov tests. In all instances where raw data were not normally distributed, square root or log transformation was applied to normalize data. Normally distributed data were then evaluated by the appropriate statistical tests (specified in the figures legends), including: parametric two-tailed Student’s t test with equal variance, one-way ANOVA or repeated measures ANOVA; followed by Tukey post hoc test to specify significance, or Chi-square test for categorical variables. P ≤ 0.05 was considered significant.

## References

[1] Brunet S, Maro B (2005). Cytoskeleton and cell cycle control during meiotic maturation of the mouse oocyte: integrating time and space. Reproduction.

[2] Solc P, Schultz RM, Motlik J (2010). Prophase I arrest and progression to metaphase I in mouse oocytes: comparison of resumption of meiosis and recovery from G2-arrest in somatic cells. Mol. Hum. Reprod..

[3] Levi M, Maro B, Shalgi R (2010). The involvement of Fyn kinase in resumption of the first meiotic division in mouse oocytes. Cell Cycle.

[4] Zheng K, Meng X, Yang Y, Yu Y (2007). Requirements of Src family kinase during meiotic maturation in mouse oocyte. Requirements of Src Family Kinase During Meiotic Maturation in Mouse Oocyte..

[5] McGinnis LK, Carroll DJ, Kinsey WH (2011). Protein tyrosine kinase signaling during oocyte maturation and fertilization. Mol. Reprod. Dev..

[6] Levi M, Maro B, Shalgi R (2010). Fyn kinase is involved in cleavage furrow ingression during meiosis and mitosis. Reproduction.

[7] Mattan L, Ruth K-K, Ruth S (2011). Regulation of division in mammalian oocytes: implications for polar body formation. Mol. Hum. Reprod..

[8] Luo J, McGinnis LK, Kinsey WH (2009). Fyn kinase activity is required for normal organization and functional polarity of the mouse oocyte cortex. Mol. Reprod. Dev..

[9] Bouniol-Baly C (1999). Differential Transcriptional Activity Associated with Chromatin Configuration in Fully Grown Mouse Germinal Vesicle Oocytes. Biol. Reprod..

[10] Zeng F, Schultz RM (2005). RNA transcript profiling during zygotic gene activation in the preimplantation mouse embryo. Dev. Biol..

[11] Watanabe T (2008). Endogenous siRNAs from naturally formed dsRNAs regulate transcripts in mouse oocytes. Nature.

[12] Kang MK, Han SJ (2011). Post-transcriptional and post-translational regulation during mouse oocyte maturation. BMB Rep..

[13] Ma J (2010). MicroRNA activity is suppressed in mouse oocytes. Curr. Biol..

[14] Cui XS, Sun SC, Kang YK, Kim NH (2013). Involvement of microRNA-335-5p in cytoskeleton dynamics in mouse oocytes. Reprod. Fertil. Dev..

[15] Feng R (2015). MiRNA-320 in the human follicular fluid is associated with embryo quality *in vivo* and affects mouse embryonic development *in vitro*. Sci. Rep..

[16] Grossman H, Shalgi R (2016). A Role of MicroRNAs in Cell Differentiation During Gonad Development. Results Probl. Cell Differ..

[17] Ninio-Many L, Grossman H, Shomron N, Chuderland D, Shalgi R (2013). microRNA-125a-3p reduces cell proliferation and migration by targeting Fyn. J. Cell Sci..

[18] Grossman H (2015). A novel regulatory pathway in granulosa cells, the LH/human chorionic gonadotropin-microRNA-125a-3p-Fyn pathway, is required for ovulation. FASEB J..

[19] Ninio-Many L (2014). MicroRNA miR-125a-3p modulates molecular pathway of motility and migration in prostate cancer cells. Oncoscience.

[20] Mogessie B, Schuh M (2014). Nuclear envelope breakdown: actin’ quick to tear down the wall. Curr. Biol..

[21] Sun Y-M, Lin K-Y, Chen Y-Q (2013). Diverse functions of miR-125 family in different cell contexts. J. Hematol. Oncol..

[22] Ducibella T, Duffy P, Reindollar R, Su B (1990). Changes in the distribution of mouse oocyte cortical granules and ability to undergo the cortical reaction during gonadotropin-stimulated meiotic maturation and aging *in vivo*. Biol. Reprod..

[23] Xu D (2007). Involvement of Fyn tyrosine kinase in actin stress fiber formation in fibroblasts. FEBS Lett..

[24] Shima T (2001). Interaction of the SH2 domain of Fyn with a cytoskeletal protein, beta-adducin. J. Biol. Chem..

[25] Vartiainen M (2008). Nuclear actin dynamics - From form to function. FEBS Lett..

[26] Belin BJ, Mullins RD (2013). What we talk about when we talk about nuclear actin. Nucleus.

[27] Sun Q-Y, Schatten H (2006). Regulation of dynamic events by microfilaments during oocyte maturation and fertilization. Reproduction.

[28] Simon DN, Zastrow MS, Wilson KL (2010). Direct actin binding to A- and B-type lamin tails and actin filament bundling by the lamin A tail. Nucleus.

[29] Haque F (2006). SUN1 interacts with nuclear lamin A and cytoplasmic nesprins to provide a physical connection between the nuclear lamina and the cytoskeleton. Mol. Cell. Biol..

[30] Superti-Furga G (1995). Regulation of the Src protein tyrosine kinase. FEBS Lett..

[31] Luo J, McGinnis LK, Kinsey WH (2010). Role of Fyn kinase in oocyte developmental potential. Reprod. Fertil. Dev..

[32] McGinnis LK, Albertini DF (2010). Dynamics of protein phosphorylation during meiotic maturation. J. Assist. Reprod. Genet..

[33] Burke B, Ellenberg J (2002). Remodelling the walls of the nucleus. Nat. Rev. Mol. Cell Biol..

[34] Güttinger S, Laurell E, Kutay U (2009). Orchestrating nuclear envelope disassembly and reassembly during mitosis. Nat. Rev. Mol. Cell Biol..

[35] Georgatos SD, Pyrpasopoulou A, Theodoropoulos Pa (1997). Nuclear envelope breakdown in mammalian cells involves stepwise lamina disassembly and microtubule-drive deformation of the nuclear membrane. J. Cell Sci..

[36] Peter M, Nakagawa J, Dorée M, Labbé JC, Nigg EA (1990). *In vitro* disassembly of the nuclear lamina and M phase-specific phosphorylation of lamins by cdc2 kinase. Cell.

[37] Heald R, McKeon F (1990). Mutations of phosphorylation sites in lamin A that prevent nuclear lamina disassembly in mitosis. Cell.

[38] Beaudouin J, Gerlich D, Daigle N, Eils R, Ellenberg J (2002). Nuclear Envelope Breakdown Proceeds by Microtubule-Induced Tearing of the Lamina. Cell.

[39] Portier N (2007). A Microtubule-Independent Role for Centrosomes and Aurora A in Nuclear Envelope Breakdown. Dev. Cell.

[40] Nüsse M, Egner HJ (1984). Can nocodazole, an inhibitor of microtubule formation, be used to synchronize mammalian cells? Accumulation of cells in mitosis studied by two parametric flow cytometry using acridine orange and by DNA distribution analysis. Cell Tissue Kinet..

[41] Salina D (2002). Cytoplasmic dynein as a facilitator of nuclear envelope breakdown. Cell.

[42] Panorchan P, Schafer BW, Wirtz D, Tseng Y (2004). Nuclear envelope breakdown requires overcoming the mechanical integrity of the nuclear lamina. J. Biol. Chem..

[43] Shao X, Li Q, Mogilner A, Bershadsky AD, Shivashankar GV (2015). Mechanical stimulation induces formin-dependent assembly of a perinuclear actin rim. Proc. Natl. Acad. Sci. USA..

[44] Hatch EM, Hetzer MW (2016). Nuclear envelope rupture is induced by actin-based nucleus confinement. J. Cell Biol..

[45] Azoury J, Lee KW, Georget V, Hikal P, Verlhac M-H (2011). Symmetry breaking in mouse oocytes requires transient F-actin meshwork destabilization. Development.

[46] Luo Y, Lee I-W, Jo Y-J, Namgoong S, Kim N-H (2016). Depletion of the LINC complex disrupts cytoskeleton dynamics and meiotic resumption in mouse oocytes. Sci. Rep..

[47] Roeder AD, Gard DL (1994). Confocal microscopy of F-actin distribution in Xenopus oocytes. Zygote.

[48] Parfenov VN (1995). Nuclear actin filaments and their topological changes in frog oocytes. Exp. Cell Res..

[49] Okada I, Fujiki S, Iwase S, Abe H (2012). Stabilization of actin filaments prevents germinal vesicle breakdown and affects microtubule organization in Xenopus oocytes. Cytoskeleton (Hoboken).

[50] Lénárt P (2003). Nuclear envelope breakdown in starfish oocytes proceeds by partial NPC disassembly followed by a rapidly spreading fenestration of nuclear membranes. J. Cell Biol..

[51] Mori M (2014). An Arp2/3 nucleated F-actin shell fragments nuclear membranes at nuclear envelope breakdown in starfish oocytes. Curr. Biol..

[52] Lee K, Song K (2007). Actin dysfunction activates ERK1/2 and delays entry into mitosis in mammalian cells. Cell Cycle.

[53] Dechat T (2008). Nuclear lamins: major factors in the structural organization and function of the nucleus and chromatin. Genes Dev..

[54] Bettinger BT, Gilbert DM, Amberg DC (2004). Actin up in the nucleus. Nat. Rev. Mol. Cell Biol..

[55] Shumaker D (2003). The nucleoskeleton: lamins and actin are major players in essential nuclear functions. Curr. Opin. Cell Biol..

[56] Rando OJ, Zhao K, Crabtree GR (2000). Searching for a function for nuclear actin. Trends Cell Biol..

[57] McGough A, Pope B, Chiu W, Weeds A (1997). Cofilin changes the twist of F-actin: implications for actin filament dynamics and cellular function. J. Cell Biol..

[58] Padmakumar VC (2004). Enaptin, a giant actin-binding protein, is an element of the nuclear membrane and the actin cytoskeleton. Exp. Cell Res..

[59] Zhen Y-Y, Libotte T, Munck M, Noegel Aa, Korenbaum E (2002). NUANCE, a giant protein connecting the nucleus and actin cytoskeleton. J. Cell Sci..

[60] Khatau SB (2009). A perinuclear actin cap regulates nuclear shape. Proc. Natl. Acad. Sci. USA..

[61] Fairley Ea, Kendrick-Jones J, Ellis Ja (1999). The Emery-Dreifuss muscular dystrophy phenotype arises from aberrant targeting and binding of emerin at the inner nuclear membrane. J. Cell Sci..

[62] Holaska JM, Kowalski AK, Wilson KL (2004). Emerin caps the pointed end of actin filaments: evidence for an actin cortical network at the nuclear inner membrane. PLoS Biol..

[63] Chapman NM, Yoder AN, Houtman JCD (2012). Non-Catalytic Functions of Pyk2 and Fyn Regulate Late Stage Adhesion in Human T Cells. PLoS One.

[64] Rongish BJ, Kinsey WH (2000). Transient Nuclear Localization of Fyn Kinase During Development in Zebrafish. Anat. Rec..

[65] Kaspar JW, Jaiswal AK (2011). Tyrosine phosphorylation controls nuclear export of Fyn, allowing Nrf2 activation of cytoprotective gene expression. FASEB J..

[66] Levi M, Ghetler Y, Shulman A, Shalgi R (2013). Morphological and molecular markers are correlated with maturation-competence of human oocytes. Hum. Reprod..

[67] Reut T-M, Mattan L, Dafna T, Ruth K-K, Ruth S (2007). The role of Src family kinases in egg activation. Dev. Biol..

[68] Schuh M (2011). An actin-dependent mechanism for long-range vesicle transport. Nat. Cell Biol..

